# Physical activity and cognitive function among older adults: Evidence and Intervention

**DOI:** 10.1093/geroni/igaf122.2226

**Published:** 2025-12-31

**Authors:** Yanan Luo

**Affiliations:** Peking University School of Public Health, Beijing, Beijing, China (People’s Republic)

## Abstract

Physical activity is an important modifiable risk factor for cognitive decline and dementia in older adults. This study aims to analyze the relationship between physical activity and the levels of cognitive function and its decline rates as well as the association between physical activity intensity, type, and cognitive function in older adults. Additionally, guided by the integrated model of health behavior, this study explores the construction and application of digital medical exercise interventions for patients with mild cognitive impairment. The main findings are as follows: firstly, older adults who engage in moderate or vigorous physical activity daily have higher cognitive function scores and the slower cognitive decline with age; secondly, moderate or vigorous physical activity also helps reduce the decline in cognitive function levels in patients with multimorbidity, significantly slowing cognitive decline in patients with cardiometabolic multimorbidity; thirdly, aerobic training and reaching 1000-1499 MET-min of physical activity per week are significantly associated with the higher cognitive function levels in older adults; fourthly, under the guidance of the integrated model of HAPA-TPB and integrating guidelines such as the ACSM exercise testing and prescription guide along with Delphi expert consultation, an exercise intervention program for elderly patients with mild cognitive impairment was developed. The intervention program showed high satisfaction and feasibility in a preliminary pilot study, and future research will further validate its effectiveness based on randomized controlled trials. The study suggests that a certain level of physical activity is crucial for preventing cognitive decline in older age and controlling dementia.

